# Navigating neurodiversity in higher education in India

**DOI:** 10.7554/eLife.104071

**Published:** 2024-10-25

**Authors:** Deepali Taneja, Poornima Viswanathan, Sahana V Rajan

**Affiliations:** 1 Jindal School of Psychology and Counselling, O.P. Jindal Global University Haryana India; 2 Centre for Neurodiversity Studies, O.P. Jindal Global University Haryana India

**Keywords:** being neurodivergent in academia, neurodiversity, equity, diversity, inclusion, higher education

## Abstract

The concept of neurodiversity embraces the idea that there are many different ways of perceiving, thinking about and responding to the world around us. Yet, many countries – including India, with its vast population – still struggle with this concept. Societal perceptions and stereotypes surrounding neurodiversity, often fuelled by ignorance and misinformation, contribute to the marginalisation and exclusion experienced by neurodivergent individuals. This article discusses the challenges and opportunities that neurodivergent students in Indian higher education face, highlighting the urgent need for systemic change to co-create a more inclusive and equitable learning environment.

Navigating the academic landscape of higher education presents unique challenges for neurodivergent students. While they often possess strengths like creativity and exceptional attention to detail, the demands of university life – from meeting deadlines to managing social interactions – can be overwhelming (Gillespie-Lynch et al., 2017).

B, a university student, captures this experience when describing the difficulties they face with deadlines: “It’s not about procrastination or disinterest. It’s about how my brain is wired. There’s no inherent reward in finishing a task unless it truly captivates me.” For B, assignments become battles against an internal clock that runs differently, often leading to missed deadlines and a subsequent spiral of self-doubt and shame.

Their experience resonates with countless neurodivergent students who struggle not due to a lack of motivation but because their brains process information and approach tasks differently (Armstrong, 2012). Many battle with prioritising assignments, managing their time effectively, or even completing everyday tasks like showering or laundry. The constant pressure of academic deadlines and exams can have a profound impact on their sleep patterns, emotional regulation and overall well-being (Syharat et al., 2023).

D, for instance, found the weight of university life, coupled with managing their mental health, unbearable. Standard accommodations felt like inadequate band-aid solutions that failed to address the root causes of their struggles. After a year of burnout culminating in a forced withdrawal, D returned to campus with a heightened awareness of the systemic challenges faced by neurodivergent students within an education system designed primarily for a neurotypical norm. Similarly, B, despite meticulous planning, constantly found themselves battling against time.

The seemingly mundane act of arriving late for class triggered intense feelings of shame and self-doubt, serving as a painful reminder of how their ADHD brain perceives time differently. These experiences highlight a pervasive struggle for recognition and understanding (Redshaw and McCormack, 2022). B, for instance, grapples with forgetfulness and time blindness, common traits among neurodivergent individuals. “Being late to class isn’t a sign of disrespect,” they explain, “but explaining forgetfulness or time blindness rarely helps. People often dismiss it as an excuse.” For D, constant alarms have become a lifeline, a tool to stay on track and manage the relentless demands of university life.

## Facing India’s social inequalities

In India, the complexities of neurodiversity are further compounded by its intersection with existing societal inequalities and the limitations of a chronically under-resourced education system (Velaskar, 2010). Within India’s patriarchal and heteronormative landscape, the pressure to conform is immense, particularly for women who may feel compelled to mask their neurodivergent traits to meet societal expectations of womanhood, marriage and motherhood (Costa, 2023). This is amplified for those already marginalised by caste, religion or economic disparity, rendering neurodivergent individuals from these communities doubly invisible.

Students of minority groups, such as individuals from religious minorities, LGBTQIA +individuals and Dalit minorities (members of the lowest class in the traditional Hindu social hierarchy) often bear the weight of both “different” minds and “unacceptable” identities, leading to isolation and a lack of support (Bhowmik and Bhattacharjee, 2024). This burden is further exacerbated by an education system that often lacks the resources, training and infrastructure to adequately address the diverse needs of its students. Standardised testing and little understanding of diverse learning needs make a neurodiversity-inclusive classroom a distant dream.

## The double burden of being a minority

There are, then, the stories that often remain untold: the student with dyslexia in a crowded, rural classroom, struggling to decipher the chalkboard scribbles while being labelled “stupid” despite their innate creative and problem-solving abilities; the Dalit student with auditory processing disorder in an under-resourced urban college, facing both caste-based discrimination and the sting of being deemed “unintelligent” for needing a quieter learning environment and information presented differently; the autistic student, overwhelmed by the sensory chaos of a bustling city college canteen, labelled “demanding” for simply requesting accommodations like noise-cancelling headphones that would enable them to learn; the queer student with social anxiety in a conservative university hostel, grappling with prejudice and isolation while navigating the already complex terrain of self-discovery and acceptance, further burdened by the social and communication differences inherent in their neurodiversity; the ADHD student in a competitive private university, battling time blindness and executive dysfunction in a pressure-cooker environment that unfairly dismisses their struggles as mere laziness. The cumulative toll of these experiences, magnified by the specific sociocultural contexts of Indian educational settings and intersecting layers of marginalisation, is devastating – leading to anxiety, depression, and a pervasive sense of unworthiness.

## The need for change

For neurodivergent students, this system emerges as a relentless obstacle course – demanding they constantly prove their worth, fight for basic accommodations and endure the exhausting charade of pretending to be someone they are not. A fear of vulnerability, of expressing needs that might be weaponised against them, highlights the systemic failures that leave neurodivergent individuals feeling unsupported and unheard.

“It’s like being trapped in a hall of mirrors,” A, a non-binary autistic person, confided, “but my reflection is nowhere to be found.” This aching feeling of invisibility is a sentiment echoed by many who share their life stories at the Diverse Minds Collective, a group organised by the Centre for Neurodiversity Studies at O.P. Jindal Global University in India (which the present authors are affiliated with). The Diverse Minds Collective aspires to be a safe haven for neurodivergent students to find support and solidarity. Within this space, the academic community (including students, staff, and faculty) shares stories of navigating a world that feels misaligned with their ways of being, often revealing how the unique challenges of being neurodivergent are amplified by deeply rooted systems of oppression. Participants collaboratively explore solutions and strategies for creating educational spaces that are truly neurodivergent-inclusive, recognizing that this requires both individual and systemic change.

Amidst all the challenges, discussions within the Diverse Minds Collective reveal a powerful and inspiring aspect of neurodivergence: the remarkable strengths and talents that emerge when restrictive systems adapt to acknowledge, value and nurture different minds. These hidden potentials, often mislabelled as deficits, can be invaluable in academic settings and beyond. Students who may struggle with traditional exams excel in open-ended research projects or critical essays. They bring unique insights and make connections others would not have considered, with their almost uncanny ability to identify patterns, dissect complex texts and synthesise information. The excellent attention to detail that many neurodivergent students exhibit can actually be a result of the sensory hypersensitivity that they experience (Baron-Cohen et al., 2009).

## Embracing differences

The problem with traditional education is that it pathologises differences. We label certain behaviours as “problems” instead of recognising them as potential strengths. It is not, however, about “fixing” students but dismantling a system built on the flawed assumption that there is only one right way to learn and be. For example, a student who initially struggled to keep up with the lectures and group discussions blossomed when assigned an independent research project on a topic that deeply interested him. His ability to hyperfocus, a common trait among those with ADHD, allowed him to focus deeply on the subject matter, producing a paper that impressed even seasoned faculty with its originality and depth of analysis. While time management and organisation can be challenging for individuals with ADHD, their capacity for hyperfocus – becoming completely absorbed in a task – can be a cognitive asset, enabling exceptional creativity, problem-solving, and productivity (Ashinoff and Abu-Akel, 2021).

The pain of invisibility and the struggle to fit into a neurotypical world can manifest in ways that leave deep scars, which in turn can affect the relationships a neurodivergent individual can have with themselves and also with others. This can lead to poor relationship choices and poor mental health as a means of coping with the overwhelming sensory and social demands of a world not built for them. The emotional toll of masking, the difficulty forming genuine connections, and the constant fear of being misunderstood can both shape identity and also become the very foundation of striving to make a change (as experienced by one of the authors of this article). For example, working hard to create a safe space where every student feels seen, valued and empowered to learn in their unique way, free from the pressure to conform to a narrow definition of “normal”.

## Choosing skills over conformity

Lesser-known conditions should also not be overlooked. For example, a student with dyscalculia who struggled with complex theory has been found to have an incredible talent for visualising narratives and translating concepts into vivid imagery in their writing. Students with dyspraxia, who may find writing or typing difficult, may compensate with powerful oral storytelling abilities. These gifts are remarkable, yet neurodivergent students still face significant barriers in a system that prioritises conformity over individual talents. We are, in essence, failing to unlock the potential that lies within our neurodivergent students. True inclusion must start with moving away from such a deficit-based perspective to one that recognises and nurtures their unique strengths and talents.

This recognition of inherent worth and potential also forms the foundation of the Diverse Minds Collective’s approach to a neurodivergent-inclusive future in Indian higher education. It aspires to co-create solutions that acknowledge the diverse needs within the neurodivergent community and respond with person-centric solutions rather than relying on a rigid, one-size-fits-all approach. This shift requires a fundamental reimagining of learning environments, support systems and assessment methods, moving away from standardised practices that often fail to accommodate neurodivergent learning styles and strengths (Kaplan, 2016). The question then arises: how can Indian universities translate the principles of neurodiversity inclusion into tangible action? Let’s explore some specific strategies and imagine how these changes could reshape the student experience.

## Shaping the future

Imagine a university where leaders, such as Vice Chancellors and Deans, actively champion neurodiversity inclusion. This commitment would translate into concrete actions: incorporating neurodiversity awareness into their mandates for teaching effectiveness, organising workshops for faculty to deepen their understanding of neurodivergent learning styles, and actively promoting the use of diverse assessment methods that better reflect the talents and abilities of all students.

A university should enable neurodivergent individuals to be adequately represented in various decision-making bodies and committees, ensuring their voices are heard, and their perspectives are considered in shaping university policies and practices.

Universities should be invested in the emotional intelligence and well-being of their students, recognising that these skills are essential for success in all aspects of life. This commitment would manifest in a culture of consent and respect, with emotional intelligence training integrated into the core curriculum. Courses would be designed, delivered, and assessed through inclusive pedagogies that acknowledge and accommodate diverse learning styles (Hamilton and Petty, 2023).

Universities ought to proactively support students in seeking diagnoses and accessing appropriate accommodations, recognising that this process can be fraught with challenges and requires institutional support to navigate effectively. This could involve streamlining the process of requesting accommodations, providing clear information about available resources, and even offering financial assistance for diagnostic assessments (Weis et al., 2016). Furthermore, universities could partner with independent bodies, such as NGOs specialising in neurodevelopmental assessments and support services, to ensure that students have access to a wider range of resources and expertise.

**Figure 1.**
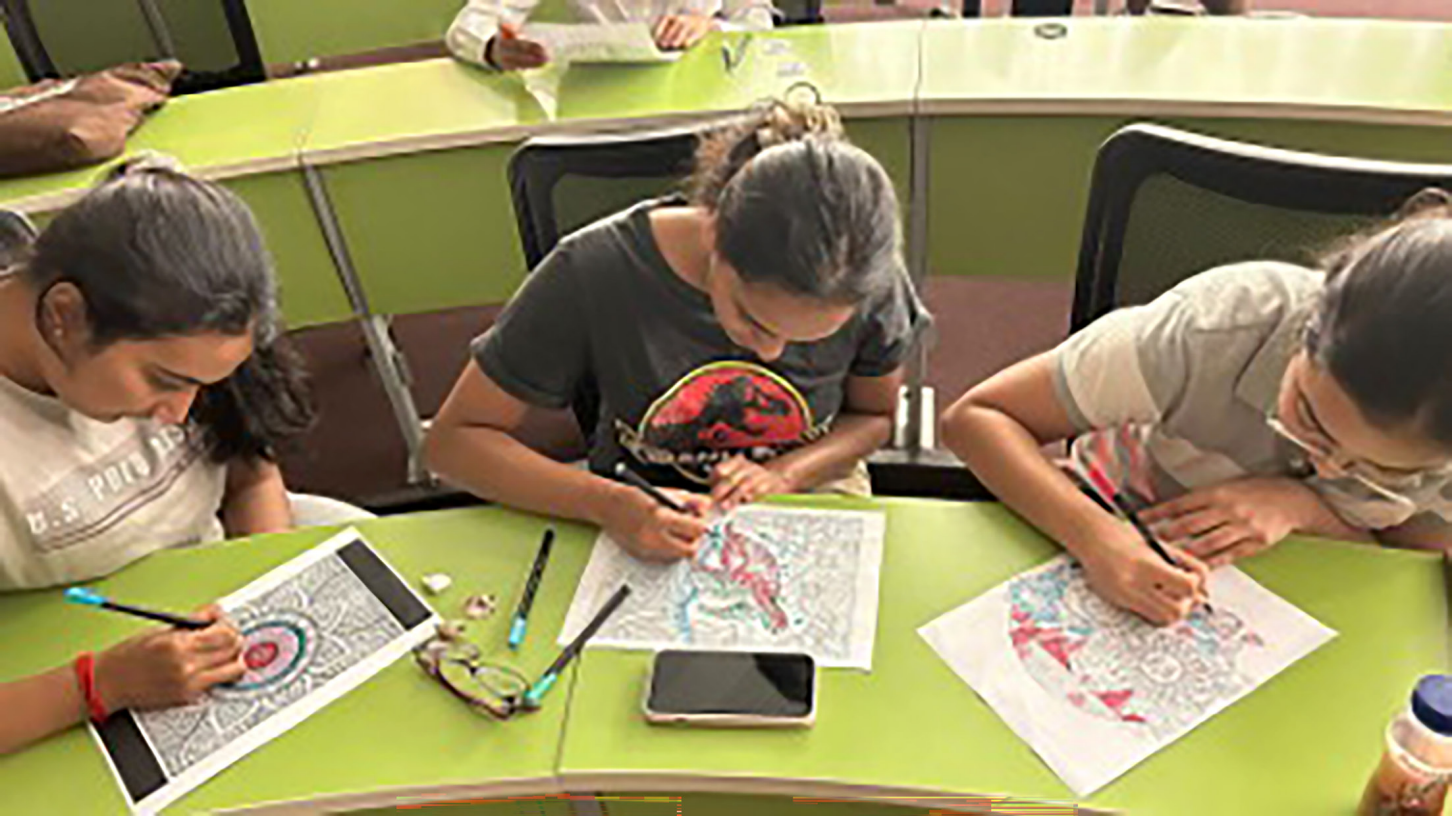
Students engaging in calming activities at an event organized by the Centre for Neurodiversity Studies at O.P. Jindal Global University.

Classrooms equipped with adjustable lighting, flexible seating arrangements, and readily available assistive technologies like text-to-speech software and noise-cancelling headphones would allow students to personalise their learning environment based on their individual sensory needs and preferences, creating a more welcoming and accessible space for all learners (Tomlinson, 2014). Imagine stepping into a university library where, alongside the traditional rows of silent study desks, you find designated quiet zones specifically designed for students with sensory sensitivities (Figure 1). These spaces would feature soft lighting, comfortable seating, and noise-reducing materials to minimise distractions and create a calming atmosphere conducive to focus and concentration (Munshaw, 2024).

Image: Sahana V Rajan.

A university needs to recognise the importance of mentorship and peer support in building a sense of belonging for neurodivergent students. Neurodivergent students can be paired with faculty mentors who understand their unique challenges and can provide guidance and support in navigating university life. This could involve assistance with academic skills, social interactions, time management, or simply having someone to talk to who understands their experiences (Locke et al., 2024).

**Figure 2.**
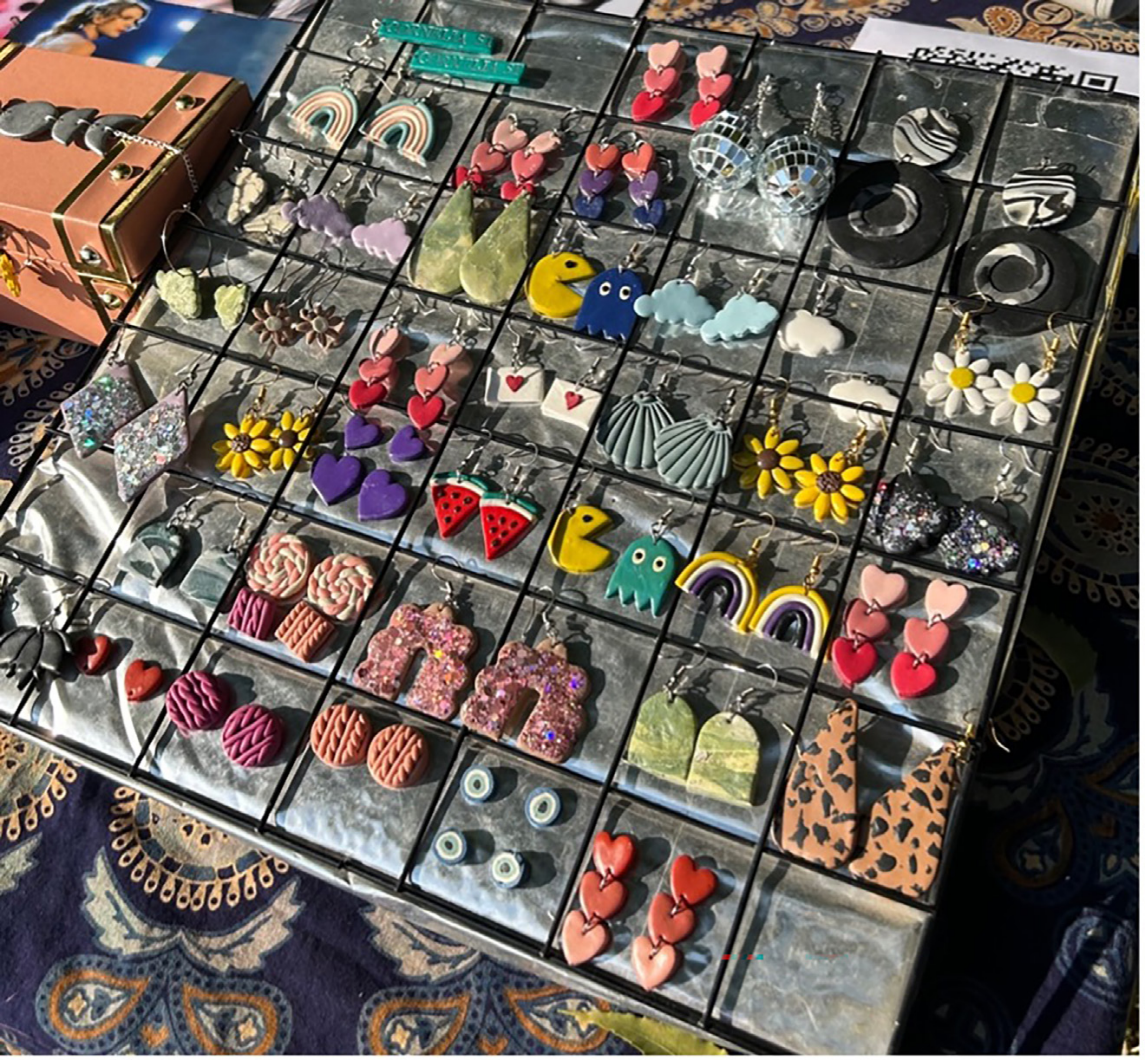
These clay creations and artworks – made by a neurodivergent student, Banipreet Kaur – were presented at an exhibition at O.P. Jindal Global University.

Extracurricular activities, such as a student-led neurodiversity club, could provide a safe and welcoming space for students to connect, share their experiences, advocate for change and build a strong sense of community. Such a club could host regular meetings, workshops, and social events, creating opportunities for neurodivergent students to find support, build friendships, and develop leadership skills. It could also organise an annual Neurodiversity Fest, showcasing the talents and perspectives of neurodivergent individuals through art, music, performances and interactive exhibits, raising awareness and celebrating neurodiversity within the larger university community (Figure 2).

Image: Banipreet Kaur.

These strategies, nurtured by a culture of empathy and understanding, hold the potential to transform Indian universities into truly inclusive spaces where every student, regardless of their neurological differences, can flourish. We owe a debt of gratitude to A, B, D, and countless others who have courageously shared their stories, allowing us to imagine the path towards a more just and equitable future for neurodivergent learners. May their voices continue to inspire us as we collectively strive to create a neurodiversity-inclusive university system.
